# Multi-omics integration analysis based on plasma circulating proteins reveals potential therapeutic targets for ulcerative colitis

**DOI:** 10.3389/fmolb.2025.1686282

**Published:** 2025-11-20

**Authors:** Jihai Zhou, Wenwen Zhao, Yiping Lin, Bo Yang, Dongjie Sun, Zhu Liu

**Affiliations:** 1 Department of Gastroenterology, Shandong Provincial Maternal and Child Health Care Hospital Affiliated to Qingdao University, Jinan, China; 2 Department of Health Medicine, 900th Hospital of PLA Joint Logistic Support Force, Fuzong Clinical Medical College of Fujian Medical University, Fuzhou, China; 3 Department of Gastroenterology and Hepatology, Guizhou Aerospace Hospital, Zunyi, China; 4 Department of Digestive Diseases, 900th Hospital of PLA Joint Logistic Support Force, Fuzong Clinical Medical College of Fujian Medical University, Fuzhou, China

**Keywords:** ulcerative colitis, multi-omics integration, diagnostic biomarkers, immune infiltration analysis, regulatory network

## Abstract

**Background:**

Ulcerative colitis (UC) is a complex inflammatory bowel disease with unclear etiology and challenging molecular mechanisms. This study aims to identify potential diagnostic and therapeutic biomarkers for UC through multi-omics integrative analysis, providing new insights into its precise diagnosis and treatment.

**Methods:**

Data samples from the Gene Expression Omnibus database and protein quantitative trait loci data from genome-wide association studies were integrated to identify overlapping genes. Three machine learning (ML) algorithms were employed to screen core hub genes from these overlapping genes, followed by the construction and external validation of a diagnostic model. Single-cell sequencing data were used to explore the expression profiles of core hub genes across different cell types. Additionally, immune infiltration, functional enrichment, and regulatory networks were analyzed. Finally, the expression trends of the core hub genes were validated in a dextran sulfate sodium (DSS)-induced UC mouse model using RT-qPCR.

**Results:**

Mendelian randomization (MR) analysis identified 168 plasma proteins causally associated with UC. Differential expression analysis revealed 1,011 DEGs, and the intersection of DEGs and MR results yielded 12 overlapping genes. Four core hub genes, including EIF5A2, IDO1, CDH5, and MYL5, were identified using three ML algorithms. The nomogram model constructed with these four genes demonstrated strong predictive performance, which was further confirmed in an external validation dataset. GSEA analysis revealed that these genes are involved in various biological processes, including immune response, signal transduction, metabolism, and cellular stress. CIBERSORT immune infiltration analysis showed significant differences in immune cell infiltration between UC and normal tissues. Furthermore, a comprehensive mRNA-miRNA-lncRNA regulatory network was constructed, identifying key molecular interactions potentially driving UC pathogenesis. Single-cell RNA sequencing analysis revealed that CDH5 is primarily expressed in endothelial cells, EIF5A2 is enriched in stem cells/T cells, IDO1 is expressed in monocytes, and MYL5 is found in epithelial and endothelial cells. Finally, RT-qPCR validation in the DSS-induced UC mouse model confirmed that the expression changes of core hub genes were consistent with bioinformatics predictions.

**Conclusion:**

This study systematically identified core diagnostic genes and their regulatory networks for UC through multi-omics integration.

## 1 Introduction

Ulcerative Colitis (UC) is a chronic inflammatory bowel disease characterized by recurrent inflammation of the colonic and rectal mucosa, leading to symptoms such as abdominal pain, diarrhea, and rectal bleeding (Ungaro et al., 2017). Globally, the incidence and prevalence of UC have been on the rise in many newly industrialized countries, with reported prevalence rates in Western countries estimated to be as high as 505 cases per 100,000 population (Ng et al., 2017). The pathogenesis of UC involves a complex interplay of genetic susceptibility, environmental factors, immune dysregulation, and gut microbiota dysbiosis, all of which collectively contribute to epithelial barrier dysfunction and chronic inflammation (Santana et al., 2022). Despite advances in understanding these mechanisms, the identification of precise therapeutic targets remains challenging due to the disease’s inherent heterogeneity and the limitations of current therapies (e.g., immunosuppressants and biologics), which often fail to achieve long-term remission in a substantial proportion of patients (Cholapranee et al., 2017; Dai et al., 2023).

Despite rapid advancements in multi-omics technologies, existing studies primarily focus on single molecules or single pathways, failing to systematically integrate interactions at the multi-omics level (Zhu et al., 2025). The integration of plasma quantitative trait loci (pQTL) analysis with genome-wide association studies (GWAS) has become a powerful tool for elucidating causal relationships between genetic variants and protein expression levels, with the potential to identify novel biomarkers and drug targets (Suhre et al., 2017). Recent genomic and proteomic studies have highlighted the role of circulating proteins in UC pathogenesis, providing insights into systemic inflammatory processes (Di Narzo et al., 2017; Chen et al., 2025). While GWAS has identified genetic susceptibility loci associated with UC pathogenesis, there remains a lack of systematic evidence regarding how these genetic predispositions translate into altered protein functions that directly lead to inflammation and tissue damage. Mendelian randomization (MR) approaches have further strengthened these findings by mitigating confounding factors and reverse causality, particularly when establishing associations between specific proteins and diseases (Verbanck et al., 2018). However, a truly comprehensive multi-omics investigation that systematically integrates genetic, transcriptomic, and single-cell RNA sequencing data remains crucial for elucidating the complex mechanisms underlying UC onset and progression and for pinpointing therapeutic targets.

Significant advancements in microarray technology and bioinformatics have profoundly propelled the development of the biomedical field. Leveraging their robust classification capabilities, the integration of machine learning (ML) with microarray analysis has emerged as a powerful tool for identifying novel diagnostic biomarkers (Bhandari et al., 2022). By learning intricate data patterns, ML techniques such as Random Forest (RF) and Support Vector Machine-Recursive Feature Elimination (SVM-RFE) have been successfully employed in UC research to select and validate potential serum biomarkers, demonstrating enhanced efficiency and accuracy (Ge et al., 2025; Zheng et al., 2025). However, existing studies often focus on single-omics data, lacking the comprehensive integration of diverse omics datasets, thereby restricting the scope and overall accuracy of the identified biomarkers (Zheng et al., 2025). Consequently, developing multi-omics integration approaches to enhance biomarker diagnostic and prognostic capabilities represents a critical direction in current biomedical research.

To address this gap, our study systematically integrates publicly available datasets, including Gene Expression Omnibus (GEO) microarray data, pQTL data from large cohorts, GWAS summary statistics for UC, and single-cell RNA sequencing data. Furthermore, a multifaceted methodological approach is employed, encompassing MR, various ML algorithms, nomogram model construction, immune infiltration analysis, and experimental validation. The overarching aim is to identify key genes with causal roles in the pathogenesis of UC, offering potential strategies for its precise treatment.

## 2 Materials and methods

### 2.1 Data sources

The GEO database is a widely used functional genomics repository for storing and retrieving high-throughput gene expression data, chips, and microarray information. Microarray data of three datasets were acquired from the GEO database (http://www.ncbi.nlm.nih.gov/geo/), including GSE87466, GSE92415, and GSE75214. GSE87466 (87 UC and 21 normal samples) and GSE92415 (162 UC and 21 normal samples) were used as the training sets. These two datasets were combined and batch-corrected using the “sva” package in R (Leek et al., 2012). GSE75214 (97 UC and 11 normal samples) was used as a validation set. Additionally, we obtained the GSE214695 dataset from the GEO database for single-cell sequencing data analysis, including six UC samples.

The detailed description of the datasets used in the MR analysis is provided in Supplementary Table S1. Specifically, a large-scale pQTL study involving 35,559 Icelandic individuals provided genetic association data for 4,907 circulating proteins (Ferkingstad et al., 2021). Proteomic analysis was conducted using a modified, multiplexed aptamer-based binding assay (SOMAscan version 4). Protein level variations were normalized for age- and sex-specific effects, followed by rank-inverse normal transformation to standardize the residuals. These normalized values were then used as phenotypes in GWAS conducted using BOLT-LMM linear mixed models. Additionally, summary-level GWAS data for UC were obtained from the IEU Open GWAS Project (https://gwas.mrcieu.ac.uk/datasets/) (Wu et al., 2024), with UC diagnosis based on the International Classification of Diseases (ICD) code (ICD-10: K51). However, the IEU Open GWAS database only provides summary-level statistical data and does not offer detailed information on individual samples. Since all the genetic association summary data used in this analysis are publicly available, no additional ethical approval is required for this study.

### 2.2 Exposure data

The genomic positions of pQTLs are generally located near the corresponding genes of specific proteins. Specifically, pQTLs that are close to homologous genes are termed “cis-pQTLs,” based on the assumption that these pQTL influence protein levels through the corresponding gene (Liu et al., 2025). To minimize bias from horizontal pleiotropy, we used only cis-pQTLs as instrumental variables (Peng et al., 2025). Based on the study by Ferkingstad et al. (Ferkingstad et al., 2021), we obtained genetic summary statistics related to plasma proteins (https://www.decode.com/summarydata/). These pQTL data were required to meet the following criteria: 1. show genome-wide significant associations (P < 5 × 10^−8^); 2. display independent associations (linkage disequilibrium r^2^ < 0.001); 3. be cis-pQTLs; and .4 have an F-test value greater than 10. Based on these criteria, we identified 241,653 SNPs associated with 4,288 proteins.

### 2.3 Outcome data

We obtained the UC cohort from the IEU Open GWAS Project, which originated from the Neale lab study and is based on UK Biobank data (OpenGWAS ID: ukb-a-553) (Bycroft et al., 2018; Papier et al., 2024). The sample size consists of 1,579 UC cases and 335,620 controls, with 10,894,596 single nucleotide polymorphisms (SNPs), and all participants are of European descent. The UK Biobank, established in 2006 in the United Kingdom, is internationally renowned for its extensive and openly accessible repository of biological and clinical information, making it a valuable resource for large-scale genetic and epidemiological research (Sudlow et al., 2015).

### 2.4 MR analysis

In this study, we conducted a proteome-wide MR analysis using the “TwoSampleMR” R package. The harmonize data function was employed to align effect alleles and effect sizes across datasets. To perform MR analysis, five different methods were utilized: MR Egger (Burgess and Thompson, 2017), weighted median (Bowden et al., 2016), inverse variance weighted (IVW) (Bowden et al., 2017), simple mode (Ai and Yang, 2023), and weighted mode (Hartwig et al., 2017). Among these, the IVW approach was primarily used to evaluate the causal relationship between exposure factors and outcomes. A p-value <0.05 from the IVW method was considered indicative of a significant causal association. The odds ratio (OR) was calculated to interpret the results, where OR > 1 suggested the exposure as a risk factor, while OR < 1 indicated a protective factor. To ensure the robustness of the findings, sensitivity analyses were performed, including tests for heterogeneity, horizontal pleiotropy, and leave-one-out analysis. The heterogeneity test assessed the consistency among different instrumental variables, with a p-value >0.05 indicating no substantial heterogeneity. The horizontal pleiotropy test examined whether instrumental variables influenced outcomes through pathways other than the exposure factor; a p-value >0.05 suggested the absence of horizontal pleiotropy, thus minimizing the impact of confounding factors. Finally, the leave-one-out analysis involved sequentially excluding individual instrumental variables to evaluate their influence on the overall results. Stability of the findings under this analysis confirmed the reliability and robustness of the conclusions.

### 2.5 Functional enrichment analysis

To elucidate the biological functions and signaling pathways associated with these differential genes, we utilized the R package “clusterProfiler” (Yu et al., 2012) to perform Gene Ontology (GO) and Kyoto Encyclopedia of Genes and Genomes (KEGG) enrichment analyses. GO analysis, drawing from the GO database, categorizes gene functions into three principal domains: biological processes (BP), cellular components (CC), and molecular functions (MF). KEGG pathway analysis was employed to annotate the pathways of identified or differentially expressed proteins, thereby exploring the key metabolic and signal transduction pathways in which these proteins or genes are involved.

### 2.6 Analysis of differentially expressed genes (DEGs)

To identify DEGs associated with UC, the “limma” R package (Ritchie et al., 2015) was employed, using |log2 fold change (FC)| > 0.9 and adj p-value <0.05 as the thresholds (Supplementary Table S2). Visualization of DEGs was performed using the “ggplot2” (Maag, 2018) and “pheatmap” R packages, which generated volcano plots and heatmaps, respectively. The “VennDiagram” package in R was used to plot the intersection of transcriptome differential genes and MR differential genes, resulting in shared significant shared DEGs (S-DEGs).

### 2.7 Identification of hub S-DEGs via ML algorithms

Three ML algorithms, including Least Absolute Shrinkage and Selection Operator (LASSO), SVM-RFE, and RF, were employed to screen hub S-DEGs. LASSO analysis was implemented with the R package “glmnet” (Duan et al., 2016) with 10 cross-validations to screen the optimal tuning parameter (λ). The SVM-RFE algorithm was utilized to select the point with the smallest cross-validation error to determine the variable through packages “e1071” and “caret” (Lin et al., 2017). The package “randomForest” was employed to develop a random forest model, and the top 5 S-DEGs with MeanDecreaseGini scores were chosen (Ishwaran and Kogalur, 2010). Finally, the genes obtained by intersecting the three ML algorithms with each other were regarded as the signature genes.

### 2.8 Construct a diagnostic nomogram and ROC analysis

The nomogram model was constructed using the “rms” package of R. Individual “Points” indicates the score of each candidate gene, and “Total Points” indicates the summation of all the scores of genes above (Balachandran et al., 2015). Furthermore, a calibration curve was applied to assess the predictive power of the nomogram model (Yang et al., 2020). ROC curves were visualized to present the area under the curve (AUC) for evaluating their diagnostic value. In general, an AUC of 0.5 indicates no discrimination; values between 0.7 and 0.8 are considered acceptable, 0.8–0.9 excellent, and above 0.9 outstanding.

### 2.9 Diagnostic value of hub candidate genes

The value of four hub candidate genes was evaluated via ROC curves. The external dataset GSE75214 was used to validate the ability of the prediction model to distinguish between UC and normal samples.

### 2.10 Gene set enrichment analysis (GSEA) of the key genes

The single-gene GSEA was performed using the R “GSEA” package to further investigate the potential regulatory pathways of key genes in UC. A P-value of < 0.05 was set as the threshold for significant enrichment.

### 2.11 Immune cell infiltration and correlation analysis

The CIBERSORT algorithm was used to assess the relative abundance of 22 lymphocyte subtypes in each normal and UC sample (Newman et al., 2019). Subsequently, the relationship between hub candidate genes in UC and immune cells was evaluated by Spearman correlation analysis.

### 2.12 GeneMANIA network and mRNA-miRNA-lncRNA network construction

For the functional association analysis, we examined key genes by creating protein-protein interaction (PPI) networks using the GeneMANIA platform (http://genemania.org, accessed on 15 June 2025). This online tool helps identify gene clusters that are functionally related by integrating various types of interactions, including protein–protein and protein–DNA interactions, co-expression patterns, correlations within metabolic pathways, and characteristics of spatial colocalization (Warde-Farley et al., 2010). Based on the elucidation of the functional interaction networks of key genes, we further explored their regulatory relationships at the post-transcriptional level. Initially, online tools for predicting RNA interactions, including miRanda, miRDB, and TargetScan, were employed to explore mRNA-miRNA interactions. Only interactions consistently identified by all tools were considered valid. Next, the spongescan database was used to identify miRNA-long non-coding RNA (lncRNA) interactions and validate if they were consistently recognized across databases. Based on these data, an integrated mRNA-miRNA-lncRNA regulatory network was constructed and visualized using Cytoscape 3.10.3.

### 2.13 Single-cell RNA-seq data analysis and subcellular localization

Quality control was performed before analyzing single-cell data. We preserved high-quality cells that had fewer than 20% mitochondrial genes and expressed more than 200 genes. Given the potential existence of diploid cells, cells with genes < 200 or > 5,800 were filtered out. Thereafter, the integration workflow was conducted by the Seurat pipeline (Butler et al., 2018). The remaining cells were further scaled and normalized using a linear regression model with the “Log-normalization” method, and the top 2,500 variable genes were detected by the “FindVariablFeatures” function. Subsequently, the dimensionality of the single-cell data was diminished through principal component analysis. To address batch effects across samples, the Harmony package (v1.2.0) was utilized, selecting the top 15 principal components for dimensionality reduction and clustering. Following this, UMAP was employed to project the results onto a two-dimensional plot, facilitating cell type identification. The cell types to which different clusters belonged were identified using the R package “singleR”. After, single-cell data analysis was performed to study the distribution of key genes across different cell types in UC. Finally, the subcellular localization of the biomarker proteins was also predicted based on the GENECARDS database (https://www.genecards.org) to provide a basis for understanding the changes of biomarkers in the disease process.

### 2.14 Animal experiments

The UC animal model was induced in 6–8-week-old male C57BL/6 mice under specific pathogen-free conditions following established protocols (Eichele and Kharbanda, 2017). After a 7-day acclimatization period with *ad libitum* access to food and water, 12 mice were randomly allocated to control or dextran sulfate sodium (DSS) groups (n = 6 each). The animal protocols were approved by the Institutional Animal Care and Use Committee of Yi Shengyuan Gene Technology (Tianjin) Co., Ltd. (protocol number YSY-DWLL-2024713). The DSS group received 3% DSS in drinking water for 7 days, while controls drank plain water. During the study period, mice were weighed daily and assessed for fecal occult blood and stool consistency. The Disease Activity Index (DAI) was calculated as the sum of these three scores to quantify the severity of intestinal inflammation, according to the predefined scoring criteria (Supplementary Table S3; Kim et al., 2012). On day 8, euthanasia was performed on the mice via cervical dislocation, and colon tissues were collected for RT-PCR analysis.

### 2.15 Tissue RNA Extraction and RT-qPCR

Total RNA was extracted and purified from colon tissues using TRIzol reagent (ThermoFisher Scientific, No. 15596018CN). The concentration and purity of RNA were assessed using a NanoDrop spectrophotometer (ThermoFisher Scientific). Complementary DNA was synthesized via reverse transcription using 5x Evo M-MLV RT Reaction Mix and gDNA Clean Reaction Mix (Accurate Biology, No. AG11728). The RNA input amount for each sample was 1 µg. Primers were sourced from PrimerBank (https://pga.mgh.harvard.edu/primerbank/). Quantitative real-time PCR (RT-qPCR) was performed on a Bio-Rad T100 thermal cycler, with SYBR Green Master Mix (Aibotec) used for amplification. β-actin was used as the housekeeping gene for normalization (Supplementary Table S4). Data analysis was conducted using the 2,^(-ΔΔCt)^ method, and data visualization was performed using GraphPad Prism software.

### 2.16 Statistical analyses

**FIGURE 1:**
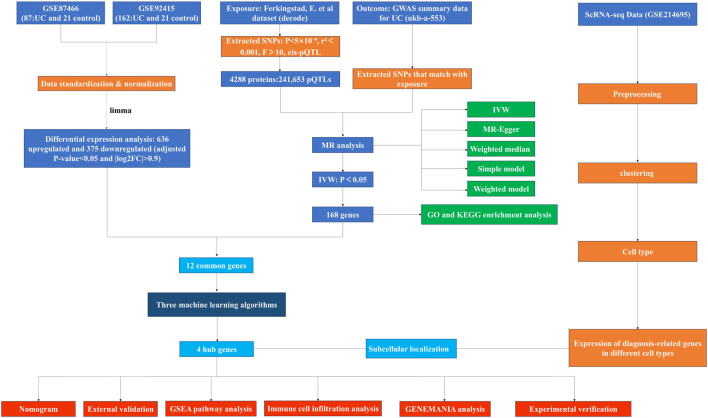
The study flowchart.

Unless otherwise specified, all statistical analyses were performed using R software (version 4.2.3). A two-tailed P-value <0.05 was considered statistically significant. The study’s workflow is illustrated in Figure 1.

## 3 Results

### 3.1 MR analysis

**FIGURE 2:**
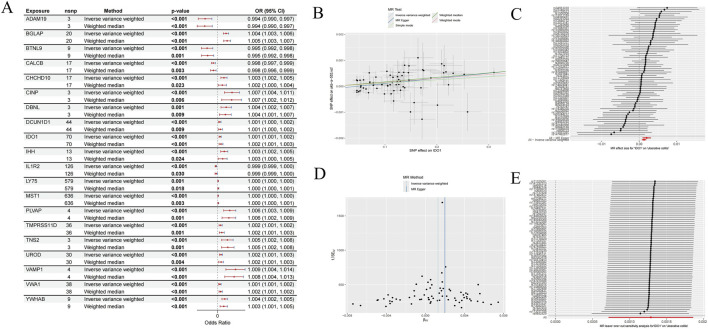
Statistical analysis of exposure effects conducted through Mendelian randomization approaches. **(A)** MR analysis results for different exposure variables, including gene loci, analysis methods, P-values, and odds ratios (randomly displayed 20 genes). **(B)** Shows the effect estimates of SNPs on outcome variables under various MR test methods. **(C)** Presents MR results of the associations between multiple SNP loci and ulcerative colitis. **(D)** Compares the effect estimates of different MR methods. **(E)** The result of the MR leave-one-out sensitivity analysis for the association between genes and outcome variables.

We performed MR analysis to evaluate the causal association between plasma circulating proteins and UC. This analysis identified 168 plasma proteins with significant causal associations with UC, of which 67 were recognized as protective factors and 101 as risk factors (Supplementary Table S5). Figure 2A, presented as a forest plot, illustrates the causal effect estimates and their 95% confidence intervals for 20 of these associated plasma proteins, intuitively demonstrating their potential direction and strength of influence on UC risk. The upregulated and downregulated status of these 20 plasma proteins has been provided in Supplementary Table S6.

To further elucidate the details and robustness of the MR analysis, we selected IDO1 as an example to comprehensively present its MR study results. The scatter plot in Figure 2B visually revealed a positive causal association between elevated IDO1 levels and increased UC risk, with consistent trends observed across fitted lines from various MR methods. Figure 2C, in the form of a forest plot, displays the estimated effect of each SNP of IDO1 on the UC outcome, indicating that the majority of SNPs showed consistent effect directions with the overall result, thereby supporting the validity of the instrumental variables. Furthermore, the funnel plot in Figure 2D exhibited a largely symmetrical shape, suggesting that the MR analysis in this study was not significantly affected by horizontal pleiotropy or publication bias. The leave-one-out sensitivity analysis (Figure 2E) further confirmed that the causal effect estimate for IDO1 on UC was highly robust and not decisively influenced by any single SNP. Detailed results of heterogeneity tests and horizontal pleiotropy tests for plasma proteins are presented in Supplementary Tables S7, S8, respectively. These comprehensive analyses robustly confirmed that all 168 plasma proteins identified by the MR approach have a significant causal relationship with UC.

### 3.2 Functional enrichment analysis

**FIGURE 3:**
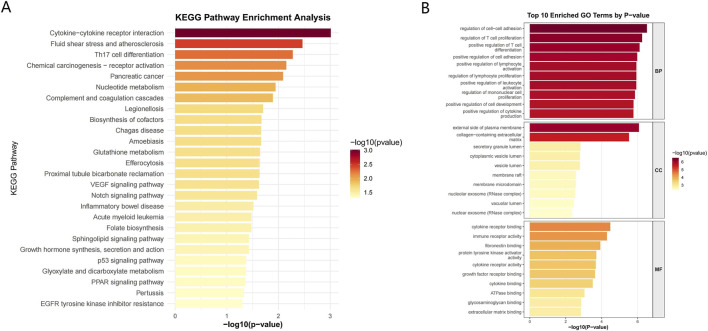
Functional enrichment analysis. **(A)** Kyoto Encyclopedia of Genes and Genomes (KEGG) pathway enrichment analysis results. **(B)** Gene Ontology (GO) enrichment analysis results.

To investigate the biological functions and signaling pathways of the 168 differential genes, we conducted GO and KEGG enrichment analyses. We identified 803 significantly enriched GO terms, comprising 723 BP, 26 CC, and 54 MF categories. Furthermore, 26 KEGG signaling pathways were also significantly enriched. KEGG pathway enrichment analysis revealed several significantly enriched pathways, primarily associated with immune and inflammatory responses, cell signaling, and disease development (Figure 3A). These pathways included cytokine-cytokine receptor interaction, fluid shear stress and atherosclerosis, Th17 cell differentiation, and chemical carcinogenesis-receptor activation. Notably, several of these enriched pathways, such as cytokine-cytokine receptor interaction and Th17 cell differentiation, are closely associated with the immune dysregulation and inflammatory cascades characteristic of UC. For BP, significantly enriched terms included regulation of cell-cell adhesion and regulation of T cell proliferation, indicating the involvement of these genes in intercellular communication and the regulation of immune cells. In terms of CC, we observed significant enrichment in terms such as the external side of the plasma membrane and the collagen-containing extracellular matrix, suggesting the contribution of these genes to extracellular structural integrity and cell surface interactions. MF analysis revealed enrichment in terms like cytokine receptor binding and immune receptor activity, emphasizing their roles in mediating immune signaling and receptor-ligand interactions (Figure 3B). These findings suggest that the identified genes play crucial roles in modulating immune responses and inflammatory processes.

### 3.3 Screening DEGs and selection of the hub genes

**FIGURE 4:**
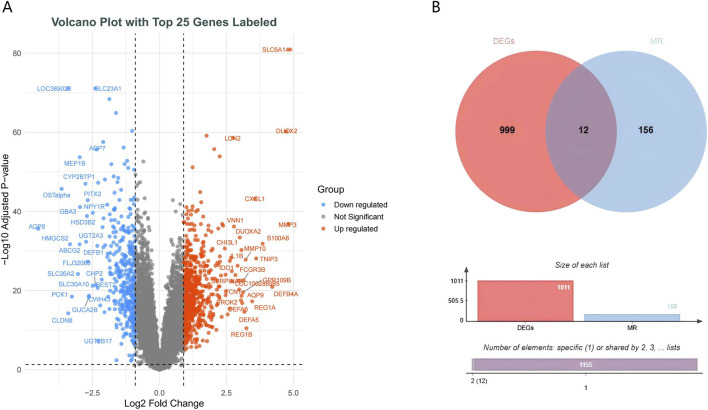
Screening of differentially expressed genes. **(A)** Volcano plot depicting gene expression changes. **(B)** Venn diagram illustrating the overlap between differentially expressed genes (DEG) and genes related to Mendelian randomization (MR).

**FIGURE 5:**
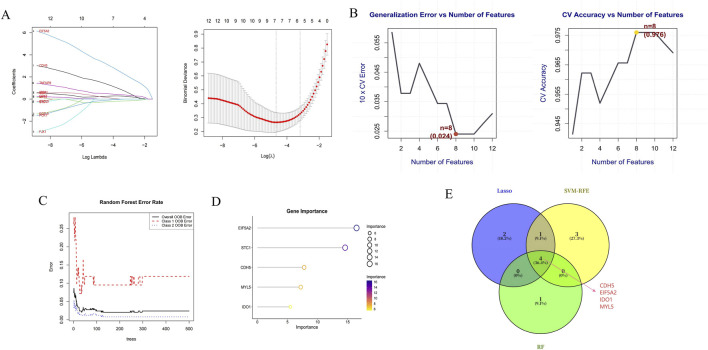
Hub gene selection. **(A)** Lasso regression plot and 10-fold cross-validation plot. **(B)** The accuracy curve and error rate curve for 5-fold cross-validation based on the SVM-RFE algorithm. **(C)** RF error rate versus the number of classification trees. **(D)** The top 5 relatively significant genes based on the RF algorithm. **(E)** Three algorithmic Venn diagram screening hub genes.

Differential expression analysis identified 636 upregulated and 375 downregulated genes (Figure 4A). To pinpoint genes common to both the transcriptome dataset and MR analysis, we intersected their respective DEGs sets, which yielded 12 S-DEGs (Figure 4B; Supplementary Table S9). ML algorithms are powerful tools for biomarker discovery and enable the identification of key genes associated with UC. To identify candidate hub S-DEGs potentially involved in UC progression, we employed three ML algorithms: LASSO regression, SVM-RFE, and RF. LASSO regression analysis identified seven candidate genes: CDH5, EIF5A2, IDO1, MMP7, MYL5, PCK1, and TNFAIP8 (Figure 5A). Eight candidate signature genes (EIF5A2, PCK1, CDH5, FJX1, IDO1, GPX7, EGFL6, and MYL5) were obtained using the SVM-RFE algorithm (Figure 5B). The RF algorithm screened the top five genes by relative importance: EIF5A2, STC1, CDH5, MYL5, and IDO1 (Figures 5C,D). Finally, a Venn diagram was used to identify the common genes among the results of these three algorithms. This intersection revealed four core signature genes: EIF5A2, CDH5, IDO1, and MYL5 (Figure 5E).

### 3.4 Construction and evaluation of a diagnostic nomogram

**FIGURE 6:**
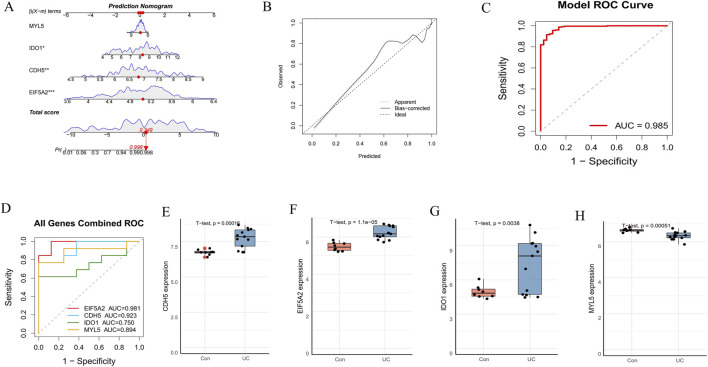
Construction and validation of a diagnostic nomogram model. **(A)** A diagnostic nomogram was developed based on the four signature genes. **(B)** Calibration curves to assess the predictive power of the nomogram. **(C)** ROC curve of the nomogram model. **(D)** ROC curve of the four hub genes in the external dataset (GSE75214). **(E–H)** The expression levels of the four hub genes in the GSE75214 dataset.

To assess the predictive capability of the identified hub genes, we constructed a diagnostic nomogram (Figure 6A). The nomogram’s predictive efficacy was then evaluated using calibration curves, which demonstrated close agreement between predicted and actual probabilities, indicating good model calibration (Figure 6B). Furthermore, ROC curve analysis revealed a high AUC of 0.985 for the nomogram, signifying its excellent diagnostic performance (Figure 6C). For external validation, individual ROC curves for these four hub genes were generated using the GSE75214 dataset (Figure 6D). All genes exhibited strong discriminative abilities, with AUC values ranging from 0.750 to 0.981. Subsequently, boxplot analyses of the GSE75214 dataset compared the expression levels of these genes between the control and UC groups (Figures 6E–H). Consistent with findings from training sets, CDH5, EIF5A2, and IDO1 were significantly upregulated, while MYL5 was significantly downregulated in the UC group compared to the control group, all demonstrating strong statistical significance (p < 0.005 for all, as indicated in figures). These comprehensive results collectively underscore the potential of EIF5A2, CDH5, IDO1, and MYL5 as promising diagnostic biomarkers for UC.

### 3.5 GSEA analysis

**FIGURE 7:**
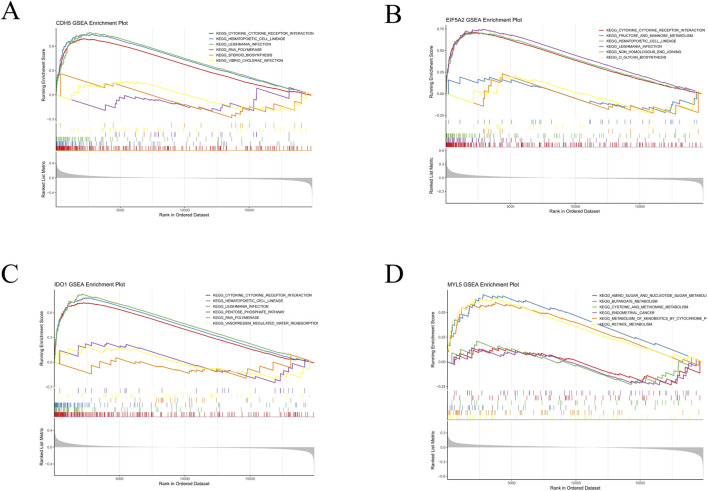
GSEA analysis of four hub genes characteristic of UC. **(A)** GSEA of CDH5 in UC. **(B)** GSEA of EIF5A2 in UC. **(C)** GSEA of IDO1 in UC. **(D)** GSEA of MYL5 in UC.

Subsequently, GSEA of the hub genes was performed, and the top three upregulated and downregulated pathways were visualized using the “clusterProfiler” package. KEGG pathway analysis revealed distinct enrichment patterns: CDH5 was significantly associated with pathways including cytokine-cytokine receptor interaction, hematopoietic cell lineage, leishmania infection, RNA polymerase, steroid biosynthesis, and *vibrio cholerae* infection (Figure 7A). EIF5A2 demonstrated enrichment in cytokine-cytokine receptor interaction, fructose and mannose metabolism, hematopoietic cell lineage, leishmania infection, non-homologous end joining, and O-glycan biosynthesis (Figure 7B). IDO1 was prominently linked to pathways such as cytokine-cytokine receptor interaction, hematopoietic cell lineage, leishmania infection, pentose phosphate pathway, RNA polymerase, and vasopressin-regulated water reabsorption (Figure 7C). MYL5 showed enrichment in pathways related to amino sugar and nucleotide sugar metabolism, butanoate metabolism, cysteine and methionine metabolism, endometrial cancer, metabolism of xenobiotics by cytochrome P450, and retinol metabolism (Figure 7D). Collectively, these findings suggest that all four biomarkers are involved in diverse biological pathways related to immune response, signal transduction, metabolism, and cellular stress, thereby highlighting their potential roles in the pathogenesis of UC.

### 3.6 Immune infiltration analysis

**FIGURE 8:**
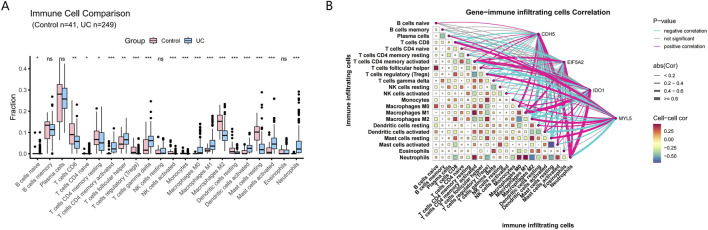
Analysis of immune cell infiltration. **(A)** Differentially expressed immune cells between control and UC samples. **(B)** Correlation analysis between immune cells and the 4 hub genes.

The CIBERSORT algorithm calculated the infiltration proportion of 22 immune cells for each sample. The result showed that the proportions of naive B cells, naive CD4 T cells, CD4 memory T cells (activated), follicular helper T cells, gamma delta T cells, M1 macrophages, activated dendritic cells, activated mast cells, and neutrophils were upregulated in the UC samples compared with the control samples. The proportions of CD8 T cells, resting CD4 memory T cells, regulatory T cells (Tregs), activated NK cells, monocytes, M0 macrophages, M2 macrophages, resting dendritic cells, and resting mast cells were downregulated in the UC group compared with the control group (Figure 8A). This finding suggests a significant difference in the immune microenvironment between the control and UC groups. We further analyzed the relationship between four hub genes and immune cell infiltration (Figure 8B). CDH5 was positively correlated with neutrophils and M0 macrophages, while negatively correlated with resting mast cells and M2 macrophages. EIF5A2 was positively correlated with neutrophils and CD4 memory T cells (activated), while negatively correlated with resting mast cells and M2 macrophages. IDO1 was positively correlated with neutrophils and M1 macrophages and CD4 memory T cells (activated), while negatively correlated with regulatory T cells (Tregs) and M2 macrophages. MYL5 was positively correlated with M2 macrophages and regulatory T cells (Tregs), while negatively correlated with neutrophils and CD4 memory T cells (activated). These findings suggest that these hub genes may play significant roles in modulating immune cell infiltration and immune responses.

### 3.7 Functional prediction of key genes and construction of mRNA-miRNA-lncRNA regulatory network

**FIGURE 9:**
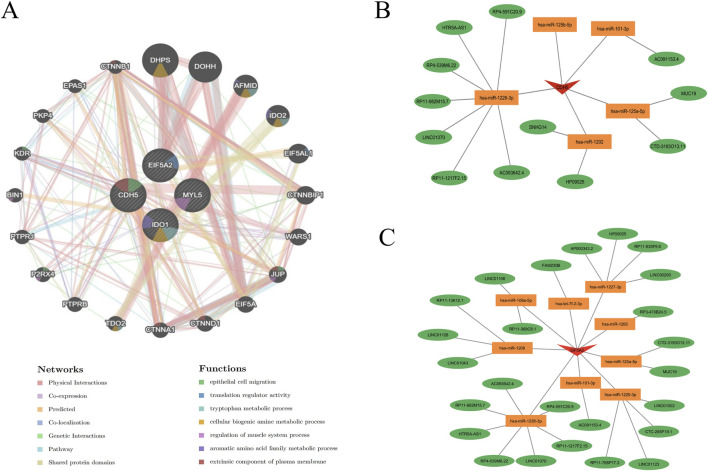
Functional prediction of key genes and construction of mRNA-miRNA-lncRNA regulatory network. **(A)** Co-expression network of hub genes predicted by GeneMANIA. **(B,C)** Construction of an mRNA-miRNA-lncRNA regulatory network using data from the miRanda, miRDB, and TargetScan databases.

To further understand the role of key genes in disease pathogenesis, these genes were submitted to the GeneMANIA database for functional and pathway prediction (Figure 9A). The results indicated that key genes were enriched in epithelial cell migration, translation regulator activity, tryptophan metabolic process, cellular biogenic amine metabolic process, regulation of muscle system process, aromatic amino acid family metabolic process, and extrinsic component of plasma membrane. Figures 9B,C illustrate mRNA-miRNA-lncRNA regulatory networks centered on CDH5 and EIF5A2, respectively. In Figure 9B, CDH5 is shown to be regulated by multiple microRNAs, including hsa-miR-125b-5p, hsa-miR-101-3p, hsa-miR-1226-3p, hsa-miR-1202, and hsa-miR-125a-5p. These microRNAs also target and regulate a shared group of lncRNAs, such as HTR5A-AS1, LINC01070, and HP09025. Similarly, Figure 9C reveals that EIF5A2 is a target of several microRNAs, including hsa-miR-1225-3p, hsa-miR-1227-3p, and hsa-miR-1226-3p, which also regulate a common set of lncRNAs, such as LINC01002, LINC01043, and LINC01106.

### 3.8 Quality control of single-cell RNA-seq data and cell annotation

**FIGURE 10:**
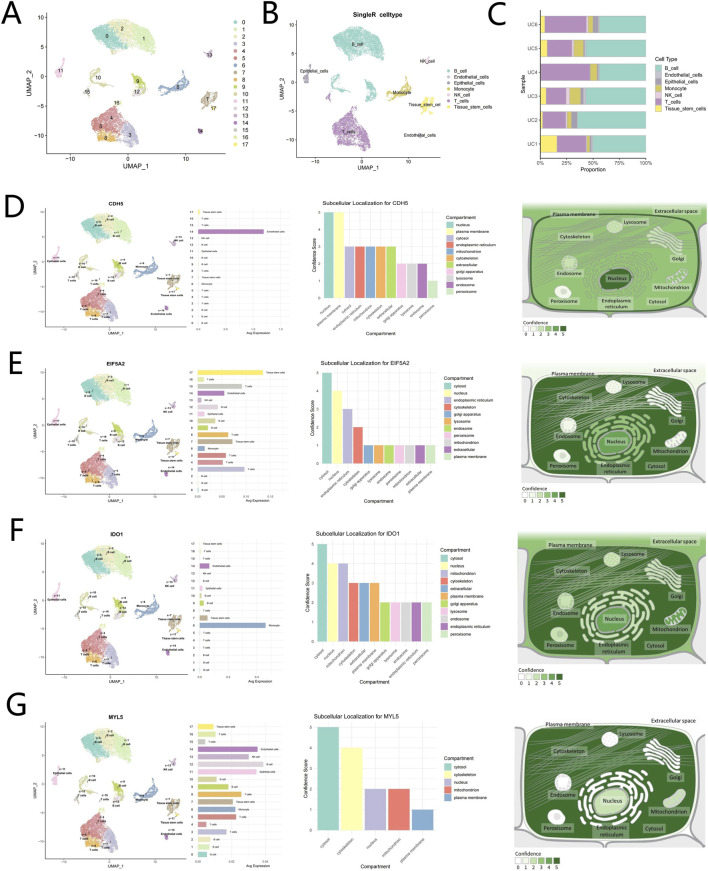
Cell annotation, single-cell expression analysis, and subcellular localization of hub genes in UC samples. **(A,B)** Cell clustering and automated annotation of clustered cells into seven cell types. **(C)** Proportional distribution of seven cell types in UC samples. **(D)** Expression distribution of CDH5 protein in UC and CDH5 subcellular localization. **(E)** Expression distribution of EIF5A2 protein in UC and EIF5A2 subcellular localization. **(F)** Expression distribution of IDO1 protein in UC and IDO1 subcellular localization. **(G)** Expression distribution of MYL5 protein in UC and MYL5 subcellular localization.

To elucidate the expression landscape of potential driver genes within the UC-associated single-cell transcriptomic data, we first conducted rigorous quality control on the single-cell dataset utilizing the Seurat package. The violin plots (Supplementary Figures S1A,B) illustrated the distributions of nFeatureRNA, nCountRNA, and percent mitochondrial before and after filtering. The selection process for the top 2,500 highly variable genes was visually represented through a scatter plot (Supplementary Figure S1C). Further analysis via scatter plots (Supplementary Figure S1D) characterized inter-variable relationships, revealing a significant negative correlation between percent mitochondrial and nCountRNA (r = −0.53). Conversely, a strong positive correlation was identified between nFeatureRNA and nCountRNA (r = 0.48). Additionally, a bubble plot (Supplementary Figure S1E) displayed the distribution of the top 25 genes contributing most significantly to the first three principal components, while a heatmap (Supplementary Figure S1F) visualized the expression patterns of these top-ranked genes across cells. The clustering analysis of the dataset identified 18 distinct clusters, which were subsequently annotated using the “SingleR” package. This process resolved the clusters into seven major cell types: B cells, endothelial cells, epithelial cells, monocytes, natural killer cells, T cells, and tissue stem cells (Figures 10A,B). The cell proportion figure illustrates the proportional distribution of seven cell types in UC samples, predominantly featuring B cells and T cells (Figure 10C). The UMAP plot illustrates elevated CDH5 expression in endothelial cell clusters, with subcellular localization analysis indicating primary distribution in the nucleus and plasma membrane (Figure 10D). EIF5A2 expression is prominently enriched in tissue stem cell and T cell clusters on the UMAP, with subcellular localization analysis indicating primary distribution in the cytosol and nucleus (Figure 10E). IDO1 expression shows enrichment in monocyte clusters via UMAP visualization, with subcellular localization analysis indicating primary distribution in the cytosol, nucleus, and mitochondria (Figure 10F). The UMAP plot reveals upregulated MYL5 expression in B cell, endothelial cell, and epithelial cell clusters, with subcellular localization analysis indicating primary distribution in the cytosol and cytoskeleton (Figure 10G).

### 3.9 Validation of hub gene expression in the experimental colitis

**FIGURE 11:**
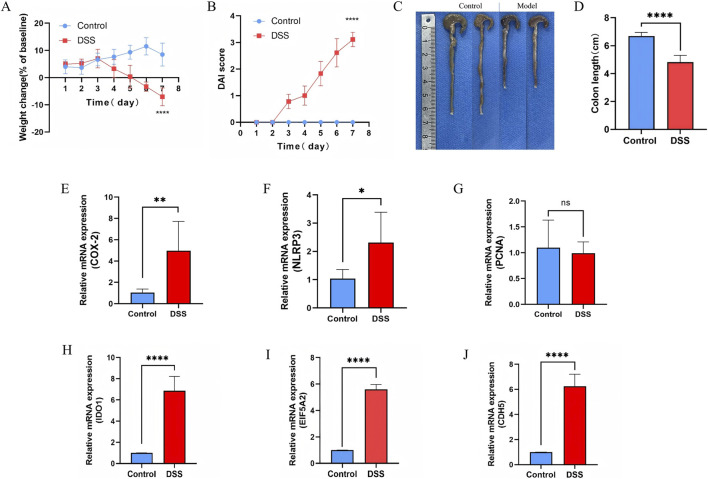
The expression of feature genes in the mouse model of UC. **(A,B)** Body weight change and DAI score in mice. **(C)** Photographs of Control colon tissue and DSS-induced colon tissue. **(D)** Changes of colon length between control and DSS-induced colon tissue. **(E)** Relative mRNA expression of COX-2 between control and DSS-induced colon tissue. **(F)** Relative mRNA expression of NLRP3 between control and DSS-induced colon tissue. **(G)** Relative mRNA expression of PCNA (Proliferating Cell Nuclear Antigen) between control and DSS-induced colon tissue. **(H)** Relative mRNA expression of IDO1 between control and DSS-induced colon tissue. **(I)** Relative mRNA expression of EIF5A2 between control and DSS-induced colon tissue. **(J)** Relative mRNA expression of CDH5 between control and DSS-induced colon tissue. ****P < 0.0001, ***P < 0.001, **P < 0.01, *P < 0.05.

To experimentally validate our bioinformatics findings, a DSS-induced UC mouse model was established. Compared to controls, DSS-treated mice exhibited significant body weight loss (Figure 11A) and progressively elevated DAI scores (Figure 11B). Clinical manifestations, including depression, dark fur, and persistent diarrhea, emerged from day 4, with perianal bleeding and soft stools evident by day 7. Additionally, the colon length was significantly reduced in the DSS group, confirming the UC model’s validity (Figures 11C,D) (Yan et al., 2009; Cao et al., 2025). In addition, we observed a significant increase in the expression of COX-2 and NLRP3, both of which are known markers of strong inflammatory response. However, there was no significant change in the expression of PCNA, a known marker of cell proliferation (Figures 11E–G). Furthermore, qRT-PCR validation confirmed the significant upregulation of EI5A2, IDO1, and CDH5 mRNA in the model group, consistent with our bioinformatics analysis (Figures 11H–J).

## 4 Discussion

UC poses significant clinical challenges due to an incomplete understanding of its genetic basis and the lack of precise therapeutic targets, often resulting in suboptimal treatment outcomes (Shimomori et al., 2025). Moreover, its heterogeneous manifestations frequently lead to diagnostic errors and delays, highlighting the urgent need for reliable early biomarkers (Sakurai and Saruta, 2023). Consequently, integrating multi-omics data to uncover causal genetic factors is essential for advancing UC research. Although prior GWAS studies have identified susceptibility loci, they typically fail to bridge genetic variants with functional protein changes and disease mechanisms at the transcriptomic and cellular levels. In this study, we implemented a comprehensive multi-omics strategy to identify and validate potential genetic drivers of UC. MR analysis of 4,288 plasma proteins from GWAS summary statistics pinpointed 168 genes with causal associations to UC, primarily enriched in immune and inflammatory pathways. Subsequent transcriptomic profiling and ML algorithms further refined this to four hub genes. Their pivotal roles were substantiated through predictive model construction, external dataset validation, and *in vivo* confirmation in a DSS-induced UC mouse model. These findings offer promising avenues for developing novel biomarkers and targeted therapies in UC.

This study identified four core genes that provide new insights into the pathological mechanisms of UC and reveal their potential roles in disease progression. EIF5A2 (eukaryotic translation elongation factor 5A2) is an important member of the eukaryotic translation elongation factor family, primarily involved in the regulation of protein translation elongation and polypeptide chain synthesis (Xiong et al., 2025). In recent years, EIF5A2 has been shown to play a critical role in various cancers, including breast cancer, liver cancer, pancreatic cancer, and gastric cancer (Bai et al., 2018; Guan et al., 2019; Wu et al., 2020; Jiang et al., 2022). However, its role in UC has not been adequately studied. For the first time, this study identified a significant upregulation of EIF5A2 in UC patients and further demonstrated its causal relationship with UC through MR analysis. This finding suggests that EIF5A2 may play an important role in the pathogenic mechanisms of UC. Additionally, a study has identified the small-molecule inhibitor SRI-011381 as a promising candidate drug for targeting EIF5A2 (Yang et al., 2023). This compound has demonstrated significant efficacy in preclinical models, highlighting the importance of developing specific inhibitors that effectively target EIF5A2 in cancer therapy. However, its role in UC remains insufficiently documented. The primary function of EIF5A2 lies in regulating protein translation elongation and polypeptide chain synthesis. Its unique translational regulatory function depends on hypusination, a highly conserved and specific post-translational modification (Suzuki et al., 2025). EIF5A2 is involved in cell proliferation, apoptosis, and inflammatory processes, as well as the regulation of transcription and RNA metabolism (Turpaev, 2018). Therefore, the upregulation of EIF5A2 may be closely associated with the activation of inflammatory signaling pathways. In this study, GSEA demonstrated that the high expression of EIF5A2 is significantly enriched in key pathways such as cytokine-cytokine receptor interaction and glucose metabolism, indicating that EIF5A2 may promote inflammation progression by regulating the activation and migration of immune cells. Particularly in the chronic inflammatory environment of UC, EIF5A2 likely exacerbates local intestinal immune imbalance by modulating T-cell function. This perspective aligns with findings in the tumor microenvironment, where EIF5A2 has been shown to promote cell migration and enhance survival signals (Wang et al., 2018). Moreover, single-cell RNA sequencing data revealed that EIF5A2 is highly expressed primarily in intestinal epithelial stem cells and T-cell populations, further suggesting its dual role in maintaining epithelial regeneration and immune homeostasis in UC. Notably, EIF5A2 has also been predicted to be a target of multiple microRNAs (e.g., hsa-miR-1225-3p and hsa-miR-1227-3p), which simultaneously regulate a group of lncRNAs, such as LINC01002 and LINC01106. This regulatory network suggests that EIF5A2 expression may be under multilayered epigenetic control, allowing precise modulation of its function in the pathological processes of UC. These findings provide a foundation for further exploration of the molecular mechanisms of EIF5A2 in UC and offer potential avenues for developing microRNA- or lncRNA-based targeted therapies. Based on these findings, our future research plans to utilize the validated small-molecule inhibitor SRI-011381 to ameliorate UC symptoms through EIF5A2 inhibition, thereby providing a novel therapeutic target for UC treatment.

In addition to EIF5A2, this study identified three other core genes, which are involved in distinct but interconnected biological processes, including immune regulation, epithelial integrity, and cellular metabolism. IDO1 plays a critical role in tryptophan metabolism and immune regulation. Its significant upregulation in UC suggests that it may regulate immune tolerance through the tryptophan-kynurenine pathway (Upadhyay et al., 2025). Furthermore, a previous study found that IDO1 promotes UC progression by inducing M1 macrophage polarization via the GRP78-XBP1 pathway, which is related to endoplasmic reticulum stress (Gao et al., 2025). In this study, we also observed that IDO1 was positively correlated with M1 macrophages and negatively correlated with M2 macrophages, suggesting that IDO1 may contribute to UC by activating M1 macrophages. Another study demonstrated that oxymatrine alleviates UC by inhibiting IDO1, thereby reducing inflammation and ferroptosis (Gao et al., 2024). Therefore, inhibiting IDO1 activity may help restore immune balance and alleviate chronic inflammation, making it a promising therapeutic target for UC intervention. Although the application of IDO1 inhibitors in UC is still in its early stages, current evidence suggests that targeting IDO1 may help restore immune balance, alleviate UC symptoms, and reduce inflammatory responses (Proietti et al., 2023; Wang et al., 2023). IDO1 inhibitors could emerge as a promising therapeutic strategy for UC, particularly when combined with other immunomodulatory therapies, potentially yielding more significant clinical benefits.

CDH5, also known as vascular endothelial cadherin, is a transmembrane protein predominantly expressed in endothelial cells and plays a critical role in maintaining vascular barrier function (Cavallaro et al., 2006). Endothelial dysfunction is a hallmark of UC progression, leading to immune cell infiltration and impaired tissue repair (Roifman et al., 2009). In this study, we also found for the first time that CDH5 is significantly upregulated in UC patients. Immune infiltration analysis indicated that CDH5 is primarily expressed in regions of neutrophil infiltration. Single-cell RNA sequencing further confirmed that CDH5 is highly expressed in endothelial cell clusters, highlighting its role in vascular remodeling during inflammation. These findings suggest that CDH5 may contribute to the inflammatory microenvironment of UC by mediating immune cell migration and endothelial activation. Additionally, the role of CDH5 in leukocyte adhesion and endothelial permeability indicates that it may be involved in the disruption of the mucosal barrier (Müller et al., 2022). Targeting CDH5 to enhance endothelial integrity and reduce immune cell infiltration could represent a novel therapeutic strategy to alleviate inflammation and promote mucosal healing.

MYL5, which encodes one of the light chains of myosin, is an essential protein for maintaining cytoskeletal dynamics and cellular contractility (Ramirez et al., 2021). Myosin is critical for maintaining epithelial structure and function, particularly in the intestinal barrier, which is frequently impaired in UC patients (Lechuga et al., 2022). A previous study reported that MYL5 expression is reduced in multiple cancers, including breast cancer, colorectal cancer, esophageal cancer, gastric cancer, head and neck cancer, and leukemia, compared to corresponding normal tissues (Lv, 2023). Similarly, our study found that MYL5 is significantly downregulated in UC patients, suggesting its potential involvement in barrier dysfunction and impaired epithelial repair. GSEA analysis showed that MYL5 is enriched in pathways related to amino sugar and nucleotide sugar metabolism, cysteine and methionine metabolism, and retinol metabolism, indicating its role in stress response and metabolic regulation. These pathways are essential for maintaining epithelial homeostasis, and reduced MYL5 expression may lead to cytoskeletal instability, increased intestinal permeability, and exacerbated inflammation. Single-cell RNA sequencing revealed that MYL5 is primarily expressed in intestinal epithelial and endothelial cells, further confirming its importance in maintaining the structural integrity of the intestinal barrier. Restoring MYL5 expression or compensating for its functional loss may provide a potential strategy to reduce intestinal permeability and prevent UC-associated inflammatory exacerbation.

The notable strength of this study lies in the comprehensive application of multi-omics approaches, which integrated MR analysis, gene expression profiling, ML algorithms, and *in vivo* validation to systematically identify and validate causal genetic factors for UC. This multi-layered research strategy not only effectively reduced the false-positive results commonly observed in single-omics studies but also significantly enhanced the reliability and reproducibility of the findings. Furthermore, the use of single-cell RNA sequencing allowed us to localize gene expression to specific cell types, providing critical insights into the cellular context of the core genes. Despite the multiple strengths of this study, certain limitations must be acknowledged. First, the MR analysis in this study relied on genetic instruments derived from GWAS data, which are predominantly based on European populations. This reliance may limit the generalizability of the findings to other ethnic groups and may not fully capture the genetic and environmental heterogeneity across populations. Future studies should focus on validating the roles of these core genes in more diverse populations to improve the universality of the findings. Additionally, the transcriptomic data used in this study were obtained from publicly available databases, lacking large-scale clinical sample validation. The inherent sample heterogeneity and batch effects may impact the robustness of the results. Future studies should incorporate multi-center samples and perform batch effect correction to further enhance data reliability. Second, although we validated the functions of three upregulated genes in a DSS-induced UC mouse model, the MYL5 gene does not exist in mice, making it impossible to validate in this model. Furthermore, this model primarily mimics acute intestinal inflammation and may not fully recapitulate the chronic and relapsing nature of human UC. Additionally, inflammation induced by DSS progresses more rapidly and severely than inflammation observed in human UC. Moreover, there are notable differences between mice and humans in terms of immune system architecture and intestinal microenvironment. Therefore, future studies should incorporate patient-derived organoid models or chronic UC animal models to further validate the roles of these genes in UC progression. Additionally, since this study heavily relies on computational predictions and bioinformatics analysis, there may be certain interpretative limitations. Therefore, future studies should incorporate more comprehensive experimental data, such as gene knockout, overexpression experiments, and protein-level investigations, to further validate the functions and mechanisms of these genes. Furthermore, the lack of clinical specimens to validate the differential expression of the core genes limits the cross-species generalizability of the results. Finally, the exact molecular mechanisms through which these core genes interact within specific signaling pathways and molecular networks in UC pathogenesis remain unclear. Future research should focus on functional studies, including gene knockout, overexpression experiments, and protein-level investigations, to further elucidate the roles of these genes and support their potential as therapeutic targets.

## 5 Conclusion

In summary, this study identified four core genes—EIF5A2, IDO1, CDH5, and MYL5—providing new insights into the genetic and molecular mechanisms underlying UC. These genes play critical roles in immune regulation, barrier integrity, and cellular metabolism. Through a multi-omics approach, we not only established the causal associations of these genes with UC but also highlighted their potential as biomarkers and therapeutic targets. Future research should focus on elucidating their specific mechanisms, validating their clinical utility, and exploring their therapeutic potential to advance the precision medicine management of UC.

## Data Availability

The datasets presented in this study can be found in online repositories. The names of the repository/repositories and accession number(s) can be found in the article/Supplementary Material.
