# Supplementary material for: Multi-omics integration analysis based on plasma circulating proteins reveals potential therapeutic targets for ulcerative colitis
Source: Front Mol Biosci. 2025 Nov 20;12:1686282. doi: 10.3389/fmolb.2025.1686282 (PMC12675271; doi:10.3389/fmolb.2025.1686282)

Supplementary Material

Supplementary Figures

Supplementary Figure 1. Preprocessing of single-cell RNA-seq data. (A, B) The violin plots illustrated the distributions of nFeatureRNA, nCountRNA, and percent.mitochondrial before and after filtering. **(C)** The selection process for the top 2500 highly variable genes was visually represented through a scatter plot. **(D)** The scatter plot demonstrates the correlations between mitochondrial gene proportion and total RNA counts, as well as between the number of detected genes and total RNA counts. **(E)** The bubble plot visualized the distribution of the top 25 genes contributing most significantly to the first three principal components. **(F)** The expression heatmap demonstrates the top 25 genes contributing to the first three principal components.

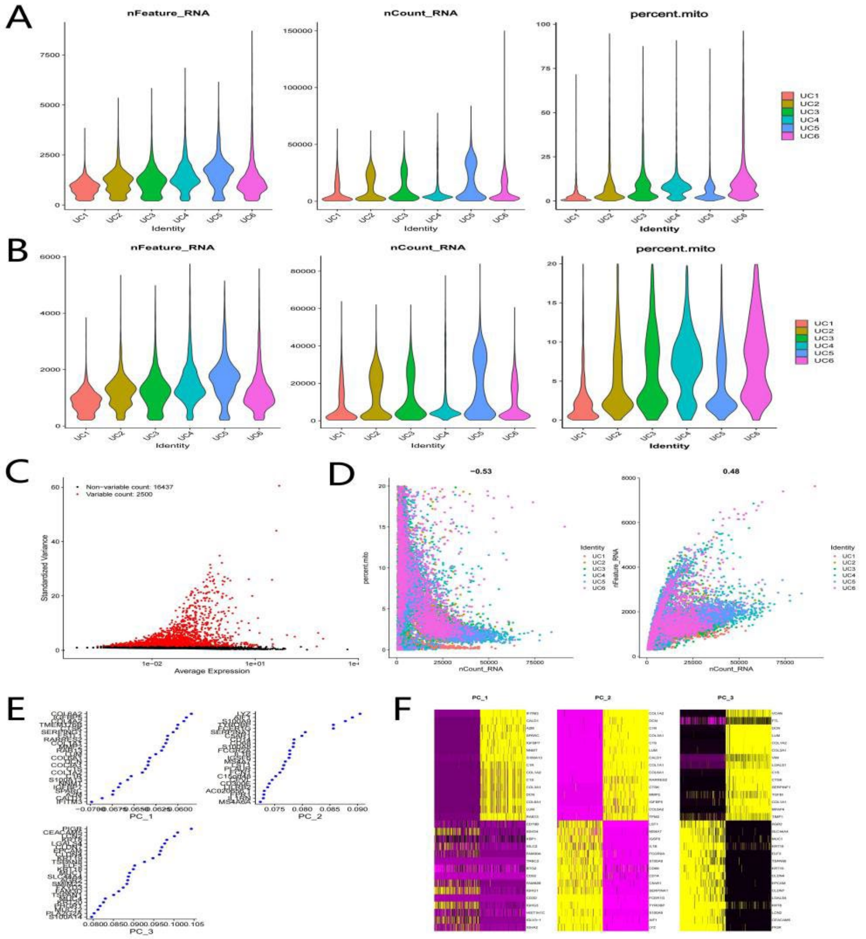

Supplement: Supplementary file 1 [file Image1.pdf]
